# Correction: Balas et al. Photodynamic Activity of TMPyP4/TiO_2_ Complex under Blue Light in Human Melanoma Cells: Potential for Cancer-Selective Therapy. *Pharmaceutics* 2023, *15*, 1194

**DOI:** 10.3390/pharmaceutics17070891

**Published:** 2025-07-09

**Authors:** Mihaela Balas, Simona Nistorescu, Madalina Andreea Badea, Anca Dinischiotu, Mihai Boni, Andra Dinache, Adriana Smarandache, Ana-Maria Udrea, Petronela Prepelita, Angela Staicu

**Affiliations:** 1 Department of Biochemistry and Molecular Biology, Faculty of Biology, University of Bucharest, 91-95 Splaiul Independentei, 050095 Bucharest, Romaniamadalina-andreea.badea@bio.unibuc.ro (M.A.B.);; 2 Laser Department, National Institute of Laser, Plasma, and Radiation Physics, 409 Atomistilor Str., 077125 Magurele, Romania; mihai.boni@inflpr.ro (M.B.); andra.dinache@inflpr.ro (A.D.); adriana.smarandache@inflpr.ro (A.S.);; 3 Research Institute of the University of Bucharest (ICUB), University of Bucharest, 90-92 Sos. Panduri, 050663 Bucharest, Romania

## Error in Figure

In the original publication [1], there was a mistake in Figure 8, as published. The image “g” from Figure 8A, representing Mel-Juso cells treated with the TMPyP4/TiO_2_ complex, was incorrectly placed in the original publication. The corrected Figure 8 appears below. The authors state that the scientific conclusions are unaffected. This correction was approved by the Academic Editor. The original publication has also been updated.

## Figures and Tables

**Figure 8:**
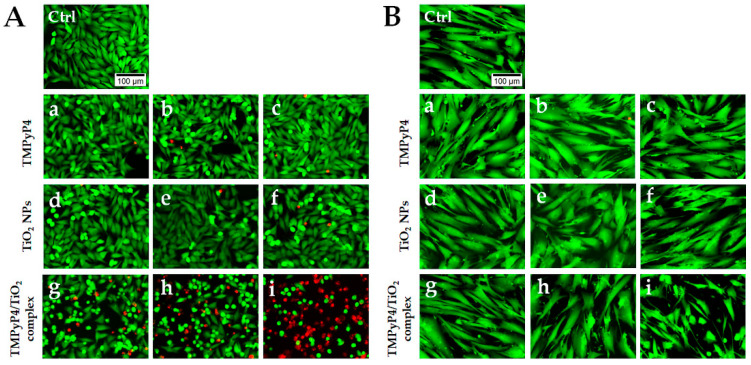
LIVE/DEAD staining on treated Mel-Juso (**A**) and CCD-1070Sk cells (**B**) after light irradiation for 7.5 min. Cells were treated with different concentrations of TMPyP4: 0.5 μg/mL (a), 0.75 μg/mL (b), and 1 μg/mL (c); TiO_2_ NPs: 20 μg/mL (d), 30.7 μg/mL (e) and 40 μg/mL (f) and TMPyP4/TiO_2_ complex: 0.5/20 μg/mL (g), 0.75/30.7 μg/mL (h), 1/40 μg/mL (i). Control cells (Ctrl) are non-treated, and light irradiated. Live cells are stained with calcein AM (green fluorescence), and dead cells with ethidium homodimer (red fluorescence). The scale bar is the same for all images and is 100 µm.
